# COVID-19—from mucosal immunology to IBD patients

**DOI:** 10.1038/s41385-021-00384-9

**Published:** 2021-02-19

**Authors:** Carl Weidinger, Ahmed Nabil Hegazy, Rainer Glauben, Britta Siegmund

**Affiliations:** 1 Department for Medicine (Gastroenterology, Infectious diseases, Rheumatology), Charité – Universitätsmedizin Berlin, corporate member of Freie Universität Berlin, Humboldt-Universität zu Berlin and Berlin Institute of Health, Campus Benjamin Franklin, Berlin, Germany; 2 Clinician Scientist Program, Berlin Institute of Health, Berlin, Germany; 3 Deutsches Rheumaforschungszentrum Berlin (DRFZ), An Institute of the Leibniz Association, Berlin, Germany

## Abstract

Viral infections with SARS-CoV-2 can cause a multi-facetted disease, which is not only characterized by pneumonia and overwhelming systemic inflammatory immune responses, but which can also directly affect the digestive system and infect intestinal epithelial cells. Here, we review the current understanding of intestinal tropism of SARS-CoV-2 infection, its impact on mucosal function and immunology and summarize the effect of immune-suppression in patients with inflammatory bowel disease (IBD) on disease outcome of COVID-19 and discuss IBD-relevant implications for the clinical management of SARS-CoV-2 infected individuals.

## Introduction

In late 2019, viral infections with the severe acute respiratory syndrome coronavirus 2 (SARS-CoV-2) emerged as a global pandemic, threatening health care systems worldwide and killing more than 1,545,723 people as of 8th of December 2020 (https://coronavirus.jhu.edu/).^1^ While most patients infected with SARS-CoV2 suffer from symptoms that are caused by infections of the upper airways including coughing, dyspnea and anosmia, it recently became evident, that Coronavirus induced disease (COVID-19) can involve multiple organs. These extra-pulmonary manifestations of COVID-19 include acute neuronal impairment such as Guillain-Barré syndrome, acute kidney injury, myocarditis, liver injury with elevated amino-transferases and disseminated thromboembolism.^2^ Thereby, COVID-19-associated organ damage is thought to be either induced via direct virus-mediated toxicity in SARS-CoV-2 infected cells, as it is observed for endothelial cells and alveolar cells,^3–5^ or indirectly provoked by overwhelming immune responses and a consecutive cytokine storm.^6,7^ In addition, disturbances in the microcirculation of various organs, caused by thromboembolic vascular damage can result in COVID-19-associated stroke, myocardial infarction, deep vein thrombosis or pulmonary embolism.^2^ The observations that ~10–60% of SARS-CoV-2 infected patients feature gastrointestinal symptoms including diarrhea, nausea, abdominal pain and anorexia furthermore highlight, that SARS-CoV2 might directly deter intestinal homeostasis via infecting intestinal epithelial cells,^8^ which might not only have important implications for the identification and disruption of possible oral-fecal transmission routes of SARS-CoV-2 infections, but which might also be relevant for the development of possible oral vaccine strategies.^9^ The fact that about 20% of patients of a Chinese cohort of 206 SARS-CoV-2 infected individuals with mild disease exclusively developed gastrointestinal symptoms,^10^ furthermore cautions to screen symptomatic patient with signs of gastroenteritis for SARS-CoV-2 infections to avoid spread in highly susceptible patient groups such as inhabitants of elderly homes or hospitalized individuals. Importantly, Livanos et al. have recently uncovered in two independent cohorts of COVID-19 patients, that patients with GI-symptoms show a decreased mortality and feature a reduced cytokine production of several pro-inflammatory cytokines such as IL-6 and lL-17.^11^

In regard of the growing number of patients with inflammatory bowel disease (IBD), that currently affect ~6.8 million individuals worldwide,^12^ a better understanding of the molecular mechanisms and consequences of intestinal SARS-CoV-2 infections is furthermore important to specifically protect IBD patients from COVID-19 associated intestinal and systemic complications. This is particularly relevant, since patients with IBD such as Crohn’s disease (CD) and ulcerative colitis (UC) frequently receive immunosuppressive therapies including antibodies blocking TNF (e.g., infliximab and adalimumab), IL-12 and IL-23 (ustekimumab) as well as integrins such as vedolizumab,^13^ which might interfere both with the establishment of proper acute anti-viral immune responses as well as with the formation of long-term immunological memory responses against SARS-CoV-2. Therefore, we will here first briefly recapitulate general aspects of viral entry and anti-viral immune responses before reviewing the current understanding of intestinal infections with SARS-CoV-2. In the second part of this review, we will then summarize the current knowledge about the specific risks that IBD patients face in case of SARS-CoV-2 infections regarding the intake of immune suppressive drugs. Here we will also discuss possible interferences of immune modulation with COVID-19-associated hyper-inflammation.

## SARS-CoV2 tropism and entry—a brief summary

The tropism and cell entry mechanisms of SARS-CoV-2 have been extensively studied in recent months and are summarized in detail elsewhere.^14^ Briefly, the entry mechanism of SARS-CoV-2 involves a two-step process where the so-called Spike (S) protein first binds to a cell surface receptor and is subsequently cleaved by a cellular protease to facilitate membrane fusion. Both, the expression of an adequate receptor on the cell surface and the presence of a host-derived protease capable of cleaving the S protein influence cellular tropism of SARS-CoV-2. For cell entry of SARS-CoV-2, angiotensin-converting enzyme 2 (ACE2) and transmembrane serine protease 2 (TMPRSS2) are the prime receptor and critical protease, respectively.^15,16^ However, there is accumulating evidence that these two proteins alone cannot explain virus tropism, especially as clinical data point to SARS-CoV-2 infection of several organs, such as the nasopharynx, bronchus, lung, esophagus and liver, where ACE2 expression could not be detected in healthy individuals.^17,18^ This observation suggests that either ACE2 expression levels vary significantly between individuals or in the course of an infection,^18^ or that SARS-CoV-2 can use alternate receptor(s) to enter certain cell types. Indeed, 28 human genes, referred to as SARS-CoV-2 and Coronavirus-associated receptors and factors, were predicted to facilitate virus entry, based on previous data.^17^ The small intestine, especially the jejunum and ileum, seems to be a susceptible organ because of the prominent co-expression of TMPRSS2 with ACE2 as well as alanine-aminopeptidase and dipeptidyl peptidase 4, both cell-surface molecules promoting virus entry into cells. These findings are in agreement with recent data derived from a small animal model of SARS-CoV-2, suggesting that SARS-CoV-2 may actively infect and replicate in the gastrointestinal tract.^19^ A further recent study demonstrated that oral SARS-CoV-2 inoculation could establish a subclinical respiratory infection accompanied by viral shedding in oral swabs and feces.^20^ Altogether, current data highlight a possible fecal-oral transmission route in experimental models and raises the question of whether fecal-oral transmission might be relevant in human COVID-19.

## General aspects of SARS-CoV2 induced immune responses

SARS-CoV-2 entry and propagation lead to innate and adaptive immune activation,^21^ which are followed by elevated serum concentrations of inflammatory cytokines and chemokines, a hallmark of severe SARS-CoV-2 infections. Here, infected cells first release chemokines and cytokines to initiate and recruit cells from both the innate and adaptive immune system to infection sites. Neutrophils and tissue-resident macrophages are among the early responders initiating inflammation and recruitment of other effector cells. Local activation of neutrophils and formation of extracellular traps are associated with dysregulated immunothrombosis promoting COVID-19 inflammatory microvascular thrombi, found in lung, kidney and heart in post-mortem analysis.^22^ Single-cell RNA sequencing analysis of bronchoalveolar lavage fluid collected from patients with COVID-19 revealed increased proportions of mononuclear phagocytes, such as macrophages, monocytes and dendritic cells.^23^ Thereby, lung residing macrophages of patients suffering from severe COVID-19, appear to preferentially recruit additional pro-inflammatory monocytic cells as well as neutrophils from the periphery in a CCR1- and CXCR3-dependent manner, whereas lung residing macrophages of patients with moderate disease course might predominantly recruit T cells by releasing CXCR3- and CXCR6-attracting chemokines,^23^ ultimately supporting a T cell-dependent clearance of SARS-CoV2.

Moreover, a recent multi-omics approach revealed a disbalance within the myeloid cell compartment in peripheral blood during COVID-19, particularly in patients with severe disease courses.^24^

Thus, profound virus-induced innate immune cell activation is commonly observed in COVID-19 and could promote SARS-CoV2-mediated immunopathology.

The induction of SARS-CoV-2-specific effector and memory T and B cell responses reflects adaptive immune cell activation and is regarded to be essential for long-term protection. A successful T cell response was doubted initially because of the observed lymphopenia with reduced CD4^+^ and CD8^+^ T cell numbers in severe COVID-19 cases. However, it is currently believed that the observed lymphopenia reflects local recruitment and migration of effector T cells to the site of infection. Indeed, CD4^+^ and CD8^+^ effector T cells, specific for SARS-CoV-2, are found in the convalescent individuals after mild COVID-19. Interestingly, these T cells recognize various SARS-CoV-2-derived peptides including viral spike, nucleoprotein and matrix as well as other viral proteins.^25–27^ This effector T cell response is accompanied by the generation of spike-specific neutralizing antibodies, memory B cells and circulating follicular T cells.^28,29^ However, T cell responses’ breadth and magnitude were significantly higher in severe compared with mild cases of COVID-19. Surprisingly, 30–50% of healthy people with no detectable SARS-CoV-2 infection also have spike-protein-specific memory CD4^+^ and CD8^+^ T cells.^25–27^ These T cell responses in healthy individuals were shown to be cross-reactive with other human coronaviruses.^30^ However, the relevance and protective capacity of such a pre-existing memory T cell response in subsequent SARS-CoV-2 exposures remain to be determined.

Moreover, an excessive inflammatory response to SARS-CoV-2, mediated by the activation of both, the innate and adaptive immune system as well as high levels of circulating cytokines and chemokines, is thought to be a key driver of disease severity and death in COVID-19 patients. Several studies have described higher plasma levels of cytokines, including but not limited to IL-6, TNF, CXCL10, TNFSF14, and oncostatin M.^31^ Therefore, dozens of immunomodulatory agents and biologics are currently examined for their use in treating COVID-19 patients, even if first studies using the IL-6R-antagonist tocilizumab failed to improve the outcome of COVID-19 patients,^32^ highlighting that, a better understanding of which particular inflammatory pathways and cell types are driving the above-described excessive inflammatory response is urgently needed to guide clinical trials in COVID-19 patients. Given that immune suppressive agents such as tyrosine kinase inhibitors, thiopurines and steroids as well blocking antibodies against TNF, IL-12/23, and integrins are frequently used in patients with IBD, a better understanding of anti-viral immune responses against SARS-CoV2 will be crucial to protect IBD-patients from potential side effects of immune suppression regarding SARS-CoV-2 infection. Vice versa, future studies will have to investigate if and how SARS-CoV2 infections might influence intestinal inflammation in IBD patients.

## Gastrointestinal symptoms in COVID-19

Why should we even consider gastrointestinal symptoms in patients infected with SARS-CoV-2? To address this question, one has to revisit the severe acute respiratory syndrome (SARS). As exemplified here with one study from 2003, 20.3% of SARS patients presented with diarrhea and 38.4% developed diarrhea during the course of disease. The same study was able to prove active viral replication within the small as well as large intestine.^33^ In line, one of the very first SARS-CoV-2 studies from China, which was published early this year, described that out of 95 patients with COVID-19, 58 cases suffered from gastrointestinal symptoms, 11.6% at admission and 49.5% during hospitalization. When endoscopy of the upper GI tract was performed, esophageal bleeding with erosions was revealed in several patients as well as ulcers in one severe patient.^34^ In a recent meta-analysis, combining 4243 patients out of 60 studies, a pooled prevalence for gastrointestinal symptoms was indicated in 17.6% of COVID-19 patients (95% CI 12.3–24.5). Remarkably, 11.8% of patients with non-severe COVID-19 and 17.1% of patients with severe COVID-19 presented with gastrointestinal symptoms.^8^ A case control study using data from a health care network in New York City found that the presence of gastrointestinal symptoms such as diarrhea and nausea was associated with a 70% higher relative risk to test positive for SARS-CoV2 mRNA in patients presenting with fever and shortness of breath.^35^ Remarkably, COVID-19 patients with GI symptoms thereby displayed a significantly lower death rate, even if the duration of their illness was prolonged.^35^

In accordance, Aghemo et al. observed in an Italian case control study that the presence of GI symptoms was associated with a better clinical outcome of COVID-19 patients,^36^ which is further supported by Livanos et al., who could demonstrate for the first time by electron microscopy that SARS-CoV2 particles can be found in intestinal epithelial cells and that COVID-19 patients with GI symptoms feature significantly decreased serum levels of IL-6 and IL-17, a reduced abundance of intestinal inflammatory dendritic cells and a lower mortality when compared to patients without GI symptoms.^11^ These observations are especially important for understanding and defining new immune regulatory networks in COVID-19, which might help to overcome immune over-activation in SARS-CoV2 infected individuals.

## Intestinal SARS-CoV2 infection

### Intestinal tropism

Recent meta-analyses of publications investigating the prevalence of SARS-CoV-2 mRNA in the stool of infected individuals revealed fecal shedding of SARS-CoV-2 RNA in 48% of patients with COVID-19,^8^ suggesting that SARS-CoV-2 might also infect intestinal epithelial cells. As recently shown by Lin et al., SARS-CoV-2 viral RNA could not only be detected in feces but also in esophageal, gastric, duodenal, ileal and rectal biopsies from two COVID-19 patients with severe disease by real time PCR.^34^ In accordance, immune-histochemical analyses of duodenal biopsies obtained from a SARS-CoV-2 infected patient revealed the presence of the SARS-CoV-2 N-protein in intestinal epithelial cells,^37^ arguing in favor of a direct tropism of SARS-CoV-2 in intestinal cells, which is furthermore underlined by observations of Sia et al., who found virus replication and expression of SARS-CoV-2 N-Protein in the small and large intestine of golden hamsters as early as 2 days after infection with SARS-CoV-2.^19^ Most importantly, Livanos et al. recently detected SARS-CoV2 particles by electron microscopy in duodenal and ileal epithelial cells in COVID-19 patients as well as SARS-CoV2 N-protein.^11^ These findings are in line with observations by Gaebler et al., who could also detect SARS-CoV2 nuclear capsid as well as viral particles by immunohistochemistry and electron microscopy in duodenal and ileal biopsies of COVID-19 patients.^38^

This concept of intestinal tropism is furthermore supported, by the finding, that human small-intestinal organoids as well as colonic organoids can be infected with SARS-CoV-2 and active viral replication of SARS-CoV-2 can be observed in-vitro in human intestinal epithelial cells.^39–41^ However, it remains unclear, whether this enteral tropism of SARS-CoV-2 is toxic to human epithelial cells and whether COVID-19 associated gastrointestinal symptoms such as diarrhea and cachexia are directly caused by virus induced epithelial damage or are secondarily triggered by excessive systemic cytokine release.

Epithelial expression of ACE2, TMPRSS2 and TMPRSS4 appear to be required for viral entry of SARS-CoV-2 into intestinal epithelial cells as the intracellular viral load is positively correlated with the expression level of ACE2 in human enterocytes. Consequentially, CRISPR-Cas9 mediated knock-down of TMPRSS2 and TRMPSS4 in duodenal enterocytes results in a reduced viral infection in-vitro.^40^ Interestingly, ACE2 can mainly be found on the luminal side of enterocytes,^42^ which in our eyes argues in favor of an oral route of infection and against a basolateral uptake of the virus via a hematologic circulation of SARS-CoV-2. This is supported by in-vitro findings from Zang et al. who observed that viral entry is ~1000 fold higher at the apical side of enterocytes when compared to virus uptake of SARS-CoV-2 via the basolateral membrane in 2-D monolayers of human duodenal cells.^40^ However, the predominant entry site of SARS-CoV2 in human intestinal epithelium has not been unambiguously identified in vivo as electron microscopy pictures of ileal and duodenal epithelial cells of SARS-CoV2 infected individuals showed potential viral particles at the basolateral surface of enterocytes. In addition, subsequent blebbing of virus containing membrane vesicles could be observed at the apical site of infected enterocytes in ileal and duodenal biopsies that were obtained from COVID-19 patients suffering from gastrointestinal symptoms.^11^

Moreover, it remains to be elucidated whether the predominant source of SARS-CoV-2 for subsequent intestinal infections are previously infected-mucosal cells in the upper airways such as pharyngeal- and nasal epithelial cells, in which very high levels of ACE2 expression^16^ and SARS-CoV-2 virus can be detected in infected individuals^43^ and which might also facilitate the oral ingestion of virus or whether SARS-CoV-2 can also primarily infect intestinal cells after oral ingestion of SARS-CoV-2 by circumventing infections of the upper airways. Despite the detection of infectious virus in the feces of SARS-CoV-2 infected golden hamsters, the transfer of virus-containing formites obtained from the cages of SARS-CoV-2 infected hamsters into the cages of uninfected hamsters only led to SARS-CoV-2 infection in 1 out of 3 hamsters.^19^ Therefore, a fecal-oral transmission is in principle possible but might only play a minor role in the transmission of SARS-CoV-2 in humans. It remains to be clarified if exclusive local intestinal infections with SARS-CoV-2 can also cause systemic viral spread and the development of COVID-19 in human patients.

It is currently not known, which factors might contribute to the gastrointestinal passage of intact virus particles, that are subsequently able to infect epithelial cells: Thus, it will be crucial to better understand if, for example, higher gastric pH levels as seen in patients treated with proton pump inhibitors (PPIs)^44^ might facilitate viral persistence in the stomach and subsequently the entry of virus in the small intestine. Likewise, Almario et al. recently described, that patients receiving PPIs, as assessed by an online survey of 53,130 American individuals, revealed a significantly increased probability for positive SARS-CoV-2 tests, when compared to individuals without current intake of PPIs.^45^ This is in contrast to observations of Le et al., investigating the correlation between oral proton pump inhibitor intake and rate of SARS-CoV-2 infections and disease severity of COVID-19 in a cohort of 132,316 Korean individuals undergoing SARS-Cov-2 testing. While the users of PPIs did not show a higher incidence of SARS-CoV-2 infections, they had a 79% higher risk to develop a severe course of COVID-19, once infected, than individuals not receiving PPIs.^46^

Further studies are required to determine which other factors such as the composition of microbiota and the transit time of ingested or intestinally secreted SARS-CoV-2 might influence the persistence of infectious SARS-CoV-2 during the passage through lower sections of the gut. In a first experiment, Zang et al. could demonstrate that stimulated human colonic fluid could efficiently inactivate SARS-CoV-2 while Rota-virus controls were still infectious after incubation with colonic fluid.^40^ Accordingly, no infectious SARS-CoV2 particles could be isolated from intestinal biopsies obtained from COVID-19 patients, in which SARS-CoV-2 N-protein expression and SARS-CoV2 mRNA had been detected in duodenal and ileal samples by immunohistochemistry and qPCR and in which virus-particles had been traced by electron microscopy, arguing against a fecal-oral transmission of SARS-CoV2 in humans as a common way of viral transmission.^11^

However, the fact that SARS-CoV-2 infected hamsters do secret infectious SARS-CoV-2 in their stool^19^ still raises the concerns of a fecal-oral transmission, especially in the context of hospitalized patients with high fecal viral load which might transmit fecal virus to medical personnel during patient-manipulations such as colonoscopies or diaper change.

So far it is not known, if other surface receptors and molecules apart from ACE2, TMPRSS2 and TMPRSS4 exist that might mediate viral infections in intestinal epithelial cells via the basolateral side. In regard of the expression of the neonatal FC receptor FcRn on enterocytes, that is involved in the basolateral-apical shuttling of immunoglobulin G (IgG) into the intestinal lumen,^47^ it seems imaginable that enterocytes could also internalize SARS-CoV-2 virus particles bound to non- or weakly neutralizing anti-SARS-CoV-2 antibodies. Such an antibody-dependent viral uptake via Fc-receptors has been shown, for example, for SARS-CoV challenged dendritic cells (reviewed by^48^).

In regard of the important role that ACE2, TMPRSS2 and TMPRSS4 play during viral uptake, it will be interesting to better delineate the mechanisms controlling their mucosal expression. Early studies, focusing on the ileal mRNA expression of *ACE2*, detected a positive correlation of ileal ACE2 expression with SARS-CoV-2 risk factors such as high BMI and age.^49^ Remarkably, inflamed tissues of CD patients revealed a lower expression of *ACE2* when compared to non-inflamed controls, suggesting that CD patients might be less prone to develop intestinal SARS-CoV-2 infections than non-inflamed individuals.^49^ Similar results could be observed by Patankar et al. who noted a significantly reduced protein expression of ACE2 in inflamed ileal biopsies of CD patients when compared to non-inflamed controls, whereas expression levels of TMPRSS2 were not altered between inflamed and non-inflamed tissue. In accordance, Suarez-Farinas et al. have recently described that ACE2 mRNA levels are significantly decreased in inflamed ileal biopsies of IBD patients, while inflamed rectal biopsies of IBD patients displayed a distinct upregulation of ACE mRNA in comparison to non-inflamed samples of IBD patients and non-IBD control patients.^50^ In contrast, Patankar et al. observed that ACE2 protein levels were comparable between inflamed colon of UC patients and non-inflamed controls.^51^

In the study of Suarez-Farinas et al., TMPRSS2 mRNA expression was significantly up regulated in inflamed ileal colonic tissue of IBD patients when compared to non-inflamed control tissue of non-IBD patients.^50^

Using DSS mouse models of intestinal inflammation, Patankar et al. could furthermore demonstrate that the ileal down regulation of ACE2 observed in CD patients might dependent on STAT1-driven repression of ACE2 via Type-1 cytokines driving CD pathogenesis, but not Type-2 cytokines, which are thought to predominantly cause UC.^51^ In line with this inflammation dependent regulation of *ACE2* expression, Burgueno et al. recorded a decreased mRNA expression levels of murine *ACE2* mRNA in intestinal epithelial cells from inflamed intestinal tissue in dextran sulfate sodium (DSS)- induced as well as in T cell transfer models of colitis.^52^

Of note, ileal *ACE2* mRNA levels significantly increased in patients with CD upon resolution of inflammation after successful treatment with the TNF blocker infliximab.^49^ This was in line with a modulation of ACE2 mRNA expression in inflamed colonic tissue of UC and CD patients, in which TNF-blocker treated patients revealed a significant upregulation of ACE2 expression when compared to non-medicated IBD groups.^50^ Taken together, these mentioned data demonstrate that both ACE2 and TRPMSS2 are dynamically regulated in a location-dependent manner in IBD. However, future studies are necessary to determine which other factors and comorbidities as well as medication might influence the expression of ACE2, TMPRSS2 and TMPRSS4 in intestinal epithelial cells, which might allow to better identify patient groups with high probability for intestinal tropism. In addition it will be important to better understand how a dynamic intestinal expression of ACE2 and TMPRSS2 might influence viral persistence and systemic spread of SARS-CoV2.

### Intestinal immune responses against SARS-CoV2

In contrast to the growing numbers of studies investigating the impact of SARS-CoV-2 infections on pulmonary  and systemic immune responses,^23–25^ few data concerning intestinal immune responses exist so far. Sia et al. have looked at local immune responses in SARS-CoV2 infected hamsters and did not observe significant lymphocyte infiltration in virus bearing epithelium or lamina propria,^19^ which was in line with findings from intestinal tissue that was obtained from SARS-CoV infected patients that did not reveal intestinal immune cell infiltration.^33^ First examinations of intestinal tissue of COVID-19 patients by immunohistochemistry and mass cytometry recently demonstrated that intraepithelial CD8^+^ lymphocytes as well as lamina propria residing CD4^+^ and CD8^+^ effector T cells are significantly expanded in SARS-CoV2 infected patients in comparison to healthy controls, whereas inflammatory dendritic cells appear significantly reduced in the lamina propria of COVID-19 patients with intestinal manifestation of COVID-19. In parallel, RNA bulk sequencing revealed a significant down regulation of pathways linked to antigen processing, T_H_17 cell differentiation and IBD. These data suggest that intestinal infection with SARS-CoV2 might alter systemic immune cell composition by shifting pro-inflammatory immune signatures toward a more favorable immune milieu. This could explain the significantly lower serum concentrations of IL-6 and IL-17 in COVID-19 patients with GI symptoms, ultimately resulting in a lower mortality when compared to COVID-19 patients without GI-symptoms symptoms.^11^

Local intestinal viral persistence might also be important for the generation and maintenance of stable anti-SARS-CoV2 antibody responses by B cells. Thus, Gaebeler et al. recently observed that SARS-CoV2 virus particles as well as SARS-CoV2 mRNA can be detected in duodenal and ileal biopsies of SARS-CoV2 patients even 3 months after initial onset of COVID-19 despite the absence of SARS-CoV2 mRNA in nasopharyngeal swabs at the time of intestinal biopsy acquisition. These data suggest, that intestinal viral pools of SARS-CoV2 might instigate a stable long-term production of neutralizing anti-viral IgA antibodies, whereas IgM and IgG levels of anti-SARS-CoV2 antibodies targeting the SARS-CoV-2 spike protein receptor binding are decreasing 6 month post infection.^38^ The longer persistence of IgA might be especially relevant for lasting immune responses since IgA dimers were found to have a potency to neutralize SARS-CoV2.^53^

**Fig. 1:**
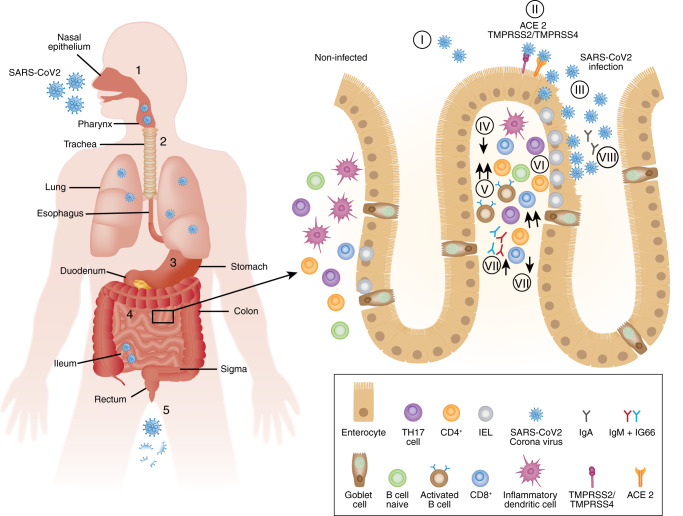
Model of intestinal SARS-CoV-2 infections. **A** Possible routes and sites of infection; 1 = infection of nasal epithelium; 2 = alternative oral ingestion of SARS-CoV-2 particles; 3 = gastric passage of infectious SARS-CoV-2 dependent on gastric pH (influenced by proton pump inhibitor intake); 4 = infection of enteric cells and active replication of virus; 5 = fecal shedding of virus or virus parts. **B** Intestinal tropism of SARS-CoV2 and subsequent changes of mucosal immune composition (I-II) = binding of SARS-CoV2 to luminal angiotensin-converting enzyme 2 (ACE2); transmembrane protease serine subtype (TMPRSS) 2- and TMPRSS4-dependent internalization of SARS-CoV-2 virus; (III) viral replication and virus dependent lysis of epithelial cells, (IV) decrease in inflammatory dendritic cells (V) infiltration with effector CD4^+^ and CD8^+^ T cells; (VI) increased abundance of IEL; (VII) decrease in IL-17 producing cells, (VIII) activation of B cells as well as formation of memory B cells through long-term persistance of SARS-CoV2 in eneterocytes and production of IgM, IgM and IgA.

Future studies will be required to understand, whether the intestinal persistence of SARS-CoV2 represents a common feature of COVID-19 or whether this can also induce long-term T cell memory responses against SARS-CoV2. The main aspects of intestinal SARS-CoV2 infections and subsequent local immune responses are summarized in Fig. 1.

## SARS-CoV2 infections in IBD patients

### Clinical symptoms

Considering the data described above, indicating that in particular the small intestine can directly be infected, the questions arose early on whether the clinical presentation in patients with IBD differs from a non-IBD cohort. In a recent population-based retrospective cohort study, IBD patients diagnosed with COVID-19 between January 20th 2020 and May 26th 2020 were compared to patients diagnosed with COVID-19 but not IBD as control group.^54^ Out of 196,403 IBD patients, 232 IBD patients diagnosed with COVID-19 were identified. The control group of non-IBD COVID-19 patients consisted of 19,776 patients. Remarkably, GI-symptoms including nausea, vomiting, diarrhea as well as abdominal pain occurred more frequently in IBD patients.^54^ A second study applied a matched cohort design and compared clinical symptoms and outcomes in COVID-19 patients with or without IBD at two New York City Hospitals. In this cohort, COVID-19 patients with IBD were matched for decade of age and gender in a 1:2 ratio to non-IBD COVID-19 patients. Here, 80 COVID-19 IBD patients were identified. In line with the data above, GI-symptoms including diarrhea and abdominal pain were significantly more frequent in COVID-19 IBD patients than in the COVID-19 non-IBD group^55^ (Table 1).

**Table 1:** Clinical symptoms with regard to the gastrointestinal tract in COVID-19 IBD and non-IBD patients.^54,55^

Clinical symptom	COVID-19 patient number	IBD COVID-19	Non-IBD COVID-19		Reference
Nausea, vomiting	IBD = 232 Control = 19.776	10.77%	4.31%	*P* < 0.01	^54^
Diarrhea	IBD = 232 Control = 19.776	8.19%	5.14%	*P* < 0.01	^54^
	IBD = 80 Control = 160	45%	19%	*P* < 0.01	^55^
Abdominal pain	IBD = 232 Control = 19.776	7.75%	2.7%	*P* < 0.01	^54^
	IBD = 80 Control = 160	20%	5%	*P* < 0.01	^55^

### Disease severity

A recent systematic literature review on the risk factors of severe COVID-19 disease, identified high age, obesity, diabetes and hypertension as key risk factors.^56^ From these data, the question arises whether or not IBD patients belong to these risk groups. Up to now, several studies have already addressed this question. In the above cited study by Singh et al., the risk of severe COVID-19 disease was similar in the IBD and the non-IBD group after Propensity Score Matching (RR 0.93, 95% CI 0.68–1.27, *P* = 0.66).^54^ In the cohort by Lukin et al. the primary outcome, a composite of death, ICU admission, or intubation was comparable (24% vs 35%, *P* = 0.352). Thus, in line with non-IBD patients, IBD patients with severe COVID-19 showed a higher age and multiple comorbidities.^54^ Accordingly, data from the Surveillance Epidemiology of Coronavirus Under Research Exclusion for Inflammatory Bowel Disease (SECURE-IBD), a large, international registry, identified increasing age, ≥2 comorbidities as well as corticosteroids, thiopurins as well as combination of thiopurins and TNF-blockers to be associated with severe COVID-19.^57^

The observation that intestinal ACE2 receptor is regulated in inflammation also raised the possibility that IBD patients might have a lower risk for enteral virus uptake. Given the fact that ACE2 and TMPRSS2 are differentially regulated in the single enteric sections and seem to be impacted by inflammatory signals, it is in our opinion to early to predict how for example the observed down regulation of ACE2 in inflamed ileal tissue of CD might affect viral entry and the clinical course of infection,^50,51^

In regard of the findings, that intestinal persistence of SARS-CoV2 might fuel the generation and maintenance of memory B cells,^38^ it is in our opinion even more difficult to interpret whether reduced intestinal ACE2 expression in IBD should be considered as a risk factor weakening anti-viral immune responses or as benefitial factor reducing the pool of SARS-CoV2.

## Impact of immune suppression on SARS-CoV2 infections in IBD patients

In regard of the frequent use of biologics and immune suppressors in patients with IBD, it appears central to delineate the effects of immune modulatory drugs in SARS-CoV2 infected IBD patients. The obvious question for IBD patients as well as physicians is whether IBD treatment can be continued during SARS-CoV-2 infection or presents a potential threat for our patients. To address this question the SECURE-IBD registry was initiated and published already first results.^57^ This first publication included 525 cases from 33 countries and focused on the effects of corticosteroids, oral salicylates and TNF-blockers, whereas numbers of other immune modulatory IBD treatments were still low at the time of this initial publication. A follow up study using the SECURE-IBD registry (now including 1439 IBD cases from 47 countries) was just recently published and now found that thiopurine, either as monotherapy or in combination with TNF-blockers, might increase the risk for severe disease course upon infection with SARS-CoV2 when compared to anti-TNF-monotherapy.^58^

Of note, all results of the SECURE-IBD registry described below should be carefully re-evaluated in independent and well-defined population-based control cohorts as the SECURE-IBD registry is a physician reported registry, that does not account for possible denominators and the respective groups are not age matched. For example, the patient group in the SECURE-IBD registry receiving TNF-blockers were significantly younger than other patient groups.^58^

With regard to the SECURE-IBD database, updates can be accessed continuously via https://covidibd.org/current-data/.

### Steroids

Despite the positive effects of dexamethasone treatment on mortality and illness severity of hospitalized COVID-19 patients with severe clinical course, that can be observed once dexamethasone is applied post infection,^59^ systemic steroid intake previous to SARS-CoV2 infection might facilitate virus replication and is associated with a higher mortality in patients with IBD (adjusted OR (aOR) 6.9; 95% CI, 1.01–1,02).^57^ In line, Singh et al. equally identified corticosteroids that were administered up to three months prior to infection as risk factor for severe disease (aOR1.60 95% CI, 1.01–2.57, *P* = 0.04). Again, patients on immune-mediated therapy in the last 12 months did not show an increased risk.^54^

### Thiopurines

As summarized above, thiopurines as well as a combination of with TNF-blocker could be associated with an increased risk of developing severe COVID-19 disease. Thus, Ungaro et al. observed a higher mortality and a prolonged disease course in IBD patients with thiopurines in the SECURE-IBD cohort particularly in comparison to TNF-blockers (aOR 4.08, 95% CI 1.73–9.61), warranting considerations to not newly begin or discontinue thiopurine therapy in IBD patients unless vaccines are broadly available.^58^

### Oral salicylates

In the first publication reporting on the results of the SECURE-IBD registry by Brenner et al., use of sulfasalazine or 5-aminosalocylate (aOR, 3.1; 95% CI, 1.3–7.7) were identified as risk factors for a severe disease course^57^ and were also associated with a higher risk for an unfavorable outcome of COVID-19 in the follow up study by Ungaro et al.^58^ However, future studies are required to investigate the mechanisms and the impact of locally applied sulfasalazine on systemic anti-viral immune responses.

### TNF-blocker

Remarkably, anti-tumor necrosis factor (anti-TNF) antibody treatment was not revealed as a risk factor (aOR, 0.9; 95% CI, 0.4–2.2) by Brenner et al.^57^ and IBD patients receiving TNF antagonists had even lower rates of severe COVID-19 compared to IBD control patients in unadjusted analyses, (1.1% vs 4.8%, *p* < 0.001),^58^ suggesting that TNF-alpha might contribute to immune over-activation and cytokine storm in COVID-19 patients.^60^ Further studies should be conducted to investigate whether TNF-blockade could be used to blunt the clinical course in patients with severe COVID-19.^61,62^

### Tyrosine kinase inhibitors

So far, no conclusive data on tyrosine kinase inhibitors in IBD has been available that would allow an assessment of their safety during the current SARS-CoV2 pandemic.

### IL-12/IL-23 blockade

So far no increased risk for severe COVID-19 could be observed for patients treated with anti-IL-12/IL-23 antibodies in the IBD-SECURE registry when compared to patients receiving anti-TNF-antagonists, suggesting that Il-12/IL-23 blockers should be considered as safe immunemodulators^58^. Moreover, IL-12/Il-23 blockade might even have benefitial effects on immune regulation in SARS-CoV2 infected patients with cytokine storm as IL-12 is a central activator of Th1 responses and  IFN-γ production.^60^

### Integrin-inhibition

To date the available literature does not indicate that integrin inhibitors such as vedolizumab would negatively affect the clinical outcome of SARS-CoV2 infected IBD patients^58^. However, future studies with bigger patient numbers will be required to specifically address possible side effects of integrin-inhibition and the development of anti-viral immune responses and immune regulation.

Taken together, it appears safe to conclude from the published data so far, that patients with IBD in general are not at an increased risk for a severe clinical course of COVID-19 and that neither TNF-, IL-12/23 – nor intergrin blockade could be identified as a risk factor, thus, emphasizing that these therapies should be continued in IBD patients.

Interestingly, Simon et al., have recently described that patients with immune mediated inflammatory diseases receiving biologics including TNF-blockade display lower levels of anti-SARS-CoV2 IgG when compared to control patients not receiving biologics. This could either mean that patients receiving biologics are protected from severe COVID-19 and thus develop less anti-SARS-CoV2 antibodies or that biologics might interfere with the generation of adequate antibody production.^63^ Therefore, we believe that future studies are required to investigate how biologics influence the generation and maintenance of anti-viral memory responses by T and B cells upon SARS-CoV2 vaccines.

An additional aspect we should consider is the recently described high incidence of thromboembolic events in patients with COVID-19.^64^ Having in mind that IBD patients in remission still harbor a twofold increased risk for developing thromboembolism,^65^ a risk that further increases in case of a flare, thromboembolism prophylaxis should be of utmost importance in hospitalized COVID-19 IBD patients.

## Aspects of SARS-CoV2 in patients with celiac disease

Although the focus of this manuscript is not on celiac disease, since it is equally affecting the small intestine, we provide a short summary here. To assess whether or not patients with celiac disease are at an increased risk of contracting COVID-19, a cross-sectional study where patients with self-reported celiac disease participated in a web-based survey. 18,022 participant provided an answer, 10,737 with self-reported celiac disease of which 65.7% reported a strict gluten-free diet. More comorbidities were observed in the celiac disease group. Overall, there was no increased risk of contracting COVID-19 in celiac disease patients.^66^ In line, in a small cohort of 21 patients with refractory celiac disease from Milan, none developed COVID-19 symptoms despite the high incidence of SARS-CoV2 in Milan during the time period of the study.^67^ Thus, at this point in line with IBD, celiac disease patients independent from gluten exposure do not seem to be at a higher risk.

## Conclusion

This SARS-CoV-2 pandemic illustrates how a close interaction between immunologists and clinicians results not only in a better understanding of disease but furthermore enables the development of potential therapeutic strategies. In addition, the insight in disease pathogenesis in parallel to data from large patient cohorts such as in SECURE-IBD provides critical data for the management of our IBD patients in the ongoing pandemic.

The observation that COVID-19 patients with GI symptoms appear to have a better clinical outcome highlight the importance that the intestinal system might play in shaping systemic immune responses and therefore it will be crucial to understand which factors and diseases such as coeliac disease, irritable bowel syndrome or colon cancer might interfere with the digestive system to modulate anti-viral immunity.

## References

[1] Dong E, Du H, Gardner L (2020). An interactive web-based dashboard to track COVID-19 in real time. Lancet Infect. Dis..

[2] Gupta A (2020). Extrapulmonary manifestations of COVID-19. Nat. Med.

[3] Ackermann M (2020). Pulmonary Vascular Endothelialitis, Thrombosis, and Angiogenesis in Covid-19. N. Engl. J. Med..

[4] Cao W, Li T (2020). COVID-19: towards understanding of pathogenesis. Cell Res..

[5] Yang L (2020). A Human Pluripotent Stem Cell-based Platform to Study SARS-CoV-2 Tropism and Model Virus Infection in Human Cells and Organoids. Cell Stem Cell.

[6] Coperchini F, Chiovato L, Croce L, Magri F, Rotondi M (2020). The cytokine storm in COVID-19: An overview of the involvement of the chemokine/chemokine-receptor system. Cytokine Growth Factor Rev..

[7] Mehta P (2020). COVID-19: consider cytokine storm syndromes and immunosuppression. Lancet.

[8] Cheung KS (2020). Gastrointestinal Manifestations of SARS-CoV-2 Infection and Virus Load in Fecal Samples From a Hong Kong Cohort: Systematic Review and Meta-analysis. Gastroenterology.

[9] Schaefer JR, Sharkova Y, Nickolaus T (2020). A SARS-CoV-2 mRNA Vaccine - Preliminary Report. N. Engl. J. Med.

[10] Han C (2020). Digestive Symptoms in COVID-19 Patients With Mild Disease Severity: Clinical Presentation, Stool Viral RNA Testing, and Outcomes. Am. J. Gastroenterol..

[11] Livanos, A. E. et al. Gastrointestinal involvement attenuates COVID-19 severity and mortality. *medRxiv* (2020).

[12] Collaborators GBDIBD (2020). The global, regional, and national burden of inflammatory bowel disease in 195 countries and territories, 1990–2017: a systematic analysis for the Global Burden of Disease Study 2017. Lancet Gastroenterol. Hepatol..

[13] Neurath MF (2019). Targeting immune cell circuits and trafficking in inflammatory bowel disease. Nat. Immunol..

[14] V’Kovski, P., Kratzel, A., Steiner, S., Stalder, H. & Thiel, V. Coronavirus biology and replication: implications for SARS-CoV-2. *Nat. Rev. Microbiol.* 1–16 (2020). 10.1038/s41579-020-00468-6 PMC7592455 33116300

[15] Hoffmann M (2020). SARS-CoV-2 Cell Entry Depends on ACE2 and TMPRSS2 and Is Blocked by a Clinically Proven Protease Inhibitor. Cell.

[16] Sungnak W (2020). SARS-CoV-2 entry factors are highly expressed in nasal epithelial cells together with innate immune genes. Nat. Med..

[17] Singh M, Bansal V, Feschotte C (2020). A Single-Cell RNA Expression Map of Human Coronavirus Entry Factors. Cell Rep..

[18] Ziegler CGK (2020). SARS-CoV-2 Receptor ACE2 Is an Interferon-Stimulated Gene in Human Airway Epithelial Cells and Is Detected in Specific Cell Subsets across Tissues. Cell.

[19] Sia SF (2020). Pathogenesis and transmission of SARS-CoV-2 in golden hamsters. Nature.

[20] Chak-Yiu Lee, A. et al. Oral SARS-CoV-2 inoculation establishes subclinical respiratory infection with virus shedding in golden Syrian hamsters. *Cell Rep. Med.* 100121 (2020). 10.1016/j.xcrm.2020.100121 PMC7508015 32984855

[21] Vabret N (2020). Immunology of COVID-19: Current State of the Science. Immunity.

[22] Nicolai L (2020). Immunothrombotic Dysregulation in COVID-19 Pneumonia Is Associated With Respiratory Failure and Coagulopathy. Circulation.

[23] Liao M (2020). Single-cell landscape of bronchoalveolar immune cells in patients with COVID-19. Nat. Med..

[24] Schulte-Schrepping J (2020). Severe COVID-19 Is Marked by a Dysregulated Myeloid Cell Compartment. Cell.

[25] Braun J (2020). SARS-CoV-2-reactive T cells in healthy donors and patients with COVID-19. Nature.

[26] Grifoni A (2020). Targets of T Cell Responses to SARS-CoV-2 Coronavirus in Humans with COVID-19 Disease and Unexposed Individuals. Cell.

[27] Peng Y (2020). Broad and strong memory CD4(+) and CD8(+) T cells induced by SARS-CoV-2 in UK convalescent individuals following COVID-19. Nat. Immunol..

[28] Cox RJ, Brokstad KA (2020). Not just antibodies: B cells and T cells mediate immunity to COVID-19. Nat. Rev. Immunol..

[29] Juno JA (2020). Humoral and circulating follicular helper T cell responses in recovered patients with COVID-19. Nat. Med.

[30] Mateus J (2020). Selective and cross-reactive SARS-CoV-2 T cell epitopes in unexposed humans. Science.

[31] Arunachalam PS (2020). Systems biological assessment of immunity to mild versus severe COVID-19 infection in humans. Science.

[32] Stone JH (2020). Efficacy of Tocilizumab in Patients Hospitalized with Covid-19. N Engl. J. Med..

[33] Leung WK (2003). Enteric involvement of severe acute respiratory syndrome-associated coronavirus infection. Gastroenterology.

[34] Lin L (2020). Gastrointestinal symptoms of 95 cases with SARS-CoV-2 infection. Gut.

[35] Nobel, Y. R. et al. Gastrointestinal Symptoms and Coronavirus Disease 2019: a Case-Control Study From the United States. *Gastroenterology* **159**, 373–375.e372 (2020). 10.1053/j.gastro.2020.04.017 PMC7152871 32294477

[36] Aghemo A (2020). COVID-19 Digestive System Involvement and Clinical Outcomes in a Large Academic Hospital in Milan, Italy. Clin. Gastroenterol. hepatology: Off. Clin. Pract. J. Am. Gastroenterological Assoc..

[37] Xiao F (2020). Evidence for Gastrointestinal Infection of SARS-CoV-2. Gastroenterology.

[38] Gaebler, C. et al. Evolution of Antibody Immunity to SARS-CoV-2. *bioRxiv*, 2020.2011.2003.367391 (2020).

[39] Lamers MM (2020). SARS-CoV-2 productively infects human gut enterocytes. Science.

[40] Zang R (2020). TMPRSS2 and TMPRSS4 promote SARS-CoV-2 infection of human small intestinal enterocytes. Sci. Immunol..

[41] Zhou J (2020). Infection of bat and human intestinal organoids by SARS-CoV-2. Nat. Med..

[42] Zhang H (2020). Specific ACE2 expression in small intestinal enterocytes may cause gastrointestinal symptoms and injury after 2019-nCoV infection. Int J. Infect. Dis..

[43] Wolfel R (2020). Virological assessment of hospitalized patients with COVID-2019. Nature.

[44] Freedberg DE, Lebwohl B, Abrams JA (2014). The impact of proton pump inhibitors on the human gastrointestinal microbiome. Clin. Lab. Med..

[45] Almario CV, Chey WD, Spiegel BMR (2020). Increased Risk of COVID-19 Among Users of Proton Pump Inhibitors. Am. J. Gastroenterol..

[46] Lee SW (2020). Severe clinical outcomes of COVID-19 associated with proton pump inhibitors: a nationwide cohort study with propensity score matching. Gut.

[47] Yoshida M (2004). Human neonatal Fc receptor mediates transport of IgG into luminal secretions for delivery of antigens to mucosal dendritic cells. Immunity.

[48] Arvin AM (2020). A perspective on potential antibody-dependent enhancement of SARS-CoV-2. Nature.

[49] Potdar, A. A. et al. Reduced expression of COVID-19 host receptor, ACE2 is associated with small bowel inflammation, more severe disease, and response to anti-TNF therapy in Crohn’s disease. *medRxiv* (2020).

[50] Suárez-Fariñas M (2020). Intestinal Inflammation Modulates the Expression of ACE2 and TMPRSS2 and Potentially Overlaps With the Pathogenesis of SARS-CoV-2-related Disease. Gastroenterology.

[51] Patankar JV (2020). The SARS-CoV-2 attachment receptor ACE2 is decreased in Crohn’s disease and regulated by microbial and inflammatory signaling. Gastroenterology.

[52] Burgueno JF (2020). Expression of SARS-CoV-2 Entry Molecules ACE2 and TMPRSS2 in the Gut of Patients With IBD. Inflamm. Bowel Dis..

[53] Wang Z (2020). Enhanced SARS-CoV-2 neutralization by dimeric IgA. Sci. Transl. Med..

[54] Singh S (2020). Risk of Severe COVID-19 in Patients with Inflammatory Bowel Disease in United States. A Multicenter Research Network Study. Gastroenterology.

[55] Lukin DJ (2020). Baseline Disease Activity and Steroid Therapy Stratify Risk of COVID-19 in Patients with Inflammatory Bowel Disease. Gastroenterology.

[56] Wolff D, Nee S, Hickey NS, Marschollek M (2021). Risk factors for Covid-19 severity and fatality: a structured literature review. Infection.

[57] Brenner EJ (2020). Corticosteroids, But Not TNF Antagonists, Are Associated With Adverse COVID-19 Outcomes in Patients With Inflammatory Bowel Diseases: Results From an International Registry. Gastroenterology.

[58] Ungaro, R. C. et al. Effect of IBD medications on COVID-19 outcomes: results from an international registry. Gut 1–8 (2020). 10.1136/gutjnl-2020-322539 PMC8136807 33082265

[59] Horby, P. et al. Dexamethasone in Hospitalized Patients with Covid-19 - Preliminary Report. *N. Engl. J. Med.* (2020). 10.1056/NEJMoa2021436 PMC7383595 32678530

[60] Fajgenbaum DC, June CH (2020). Cytokine Storm. N. Engl. J. Med..

[61] Feldmann M (2020). Trials of anti-tumour necrosis factor therapy for COVID-19 are urgently needed. Lancet.

[62] Schett G, Sticherling M, Neurath MF (2020). COVID-19: risk for cytokine targeting in chronic inflammatory diseases?. Nat. Rev. Immunol..

[63] Simon D (2020). Patients with immune-mediated inflammatory diseases receiving cytokine inhibitors have low prevalence of SARS-CoV-2 seroconversion. Nat. Commun..

[64] Wichmann D (2020). Autopsy Findings and Venous Thromboembolism in Patients With COVID-19: a Prospective Cohort Study. Ann. Intern Med.

[65] Grainge MJ, West J, Card TR (2010). Venous thromboembolism during active disease and remission in inflammatory bowel disease: a cohort study. Lancet.

[66] Zhen J (2021). The Risk of Contracting COVID-19 Is Not Increased in Patients With Celiac Disease. Clin. Gastroenterol. Hepatol..

[67] Elli L (2020). Refractory celiac disease and COVID-19 outbreak: findings from a high incidence scenario in Northern Italy. Clin. Res. Hepatol. Gastroenterol..

